# Molecular Dynamics Study of Nanoribbon Formation by Encapsulating Cyclic Hydrocarbon Molecules inside Single-Walled Carbon Nanotube

**DOI:** 10.3390/nano14070627

**Published:** 2024-04-02

**Authors:** Somayeh Eskandari, János Koltai, István László, Jenő Kürti

**Affiliations:** 1 Department of Biological Physics, Eötvös University, 1117 Budapest, Hungary; eskandari.somaye@gmail.com (S.E.); janos.koltai@ttk.elte.hu (J.K.); 2 Department of Theoretical Physics, Budapest University of Technology and Economics, 1111 Budapest, Hungary; laszlo@eik.bme.hu

**Keywords:** molecular dynamics, interatomic potentials, nanoreactors, nanotubes, nanoribbons

## Abstract

Carbon nanotubes filled with organic molecules can serve as chemical nanoreactors. Recent experimental results show that, by introducing cyclic hydrocarbon molecules inside carbon nanotubes, they can be transformed into nanoribbons or inner tubes, depending on the experimental conditions. In this paper, we present our results obtained as a continuation of our previous molecular dynamics simulation work. In our previous work, the initial geometry consisted of independent carbon atoms. Now, as an initial condition, we have placed different molecules inside a carbon nanotube (18,0): C_5_H_5_ (fragment of ferrocene), C_5_, C_5_+H_2_; C_6_H_6_ (benzene), C_6_, C_6_+H_2_; C_20_H_12_ (perylene); and C_24_H_12_ (coronene). The simulations were performed using the REBO-II potential of the LAMMPS software package, supplemented with a Lennard-Jones potential between the nanotube wall atoms and the inner atoms. The simulation proved difficult due to the slow dynamics of the H abstraction. However, with a slight modification of the parameterization, it was possible to model the formation of carbon nanoribbons inside the carbon nanotube.

## 1. Introduction

Carbon nanotubes (CNTs) are promising materials for mechanical, electrical, and optical applications. In addition, the inside of a carbon nanotube offers exciting possibilities for observing new types of chemical reactions, as shown by numerous experimental studies [1,2,3,4]. Chemical reactions take place differently in a nanotube than in free space or on the surface of a substrate. The main effect is confinement: atoms must remain close together transversely and can only move freely along the length of the tube. In addition, the atoms in the walls of the tube not only limit the available space like a hard wall, but also affect the interaction of the atoms inside. The creation of graphene nanoribbons (GNRs) within single-walled carbon nanotubes (SWCNTs) using different precursor molecules is a new approach that combines the unique properties of GNRs and SWCNTs to forge advanced nanomaterials. GNRs are particularly promising materials for nano-electronic applications because, unlike infinite graphene, they have a nonzero bandgap due to their finite width [5,6,7,8,9,10,11,12].

The formation of GNRs is facilitated by the unique, confining environment of the nanotubes’ one-dimensional space, which effectively aligns molecules such as coronene or perylene in an edge-to-edge manner, promoting their dimerization and subsequent oligomerization into extensive nanoribbons. The planar and stable nature of aromatic hydrocarbon molecules are pivotal in this synthesis.

It has recently been observed that various cyclic hydrocarbon molecules introduced into SWCNTs with a diameter of about 1.4 nm can assemble into GNRs at elevated temperatures [13,14,15]. These experimental works gave us the motivation to investigate the formation of GNRs within SWCNTs via molecular dynamics (MD) simulations.

The objective of our research was twofold: (1) we could not find any work in the literature in which MD simulation of GNR production from hydrocarbon molecules within SWCNTs has been performed (neither using DFT nor empirical functions), and we aimed to fill this gap, and (2) to test a useful empirical potential, the Brenner potential (REBO-II) for dynamic processes within SWCNTs, to our knowledge no such study can be found in the literature. The two objectives are explained in a little more detail below. 

Although there have been many experimental studies on the reactions of hydrocarbon molecules confined inside SWCNTs, we are not aware of any MD simulation studies of such processes in the literature. To avoid misunderstandings, it should be noted that many MD simulation studies have been performed on carbonaceous materials: amorphous carbon, graphite, graphene, and even carbon nanotubes and carbon nanoribbons. However, none of these have addressed the formation of GNRs inside SWCNTs [16,17,18,19,20]. We have published two MD simulation studies of GNR formation inside SWCNTs, but we placed single independent C atoms in the nanotubes as initial conditions, rather than hydrocarbon molecules [21,22]. Therefore, our first and most important goal was to continue our previous studies by using hydrocarbon molecules as precursors. We performed MD simulations for conditions as similar as possible to those of recent experimental studies.

Using empirical potentials in MD simulations is a fast and efficient method, especially for large systems and/or long simulation times. In contrast to first-principles calculations, such as DFT- or DFTB-based simulations, there is no need to diagonalize the Hamiltonian at each step, but the forces can be derived from the corresponding empirical potential functions. Carbon is a particularly difficult case due to the role of pi electrons and the competition between different possible hybridizations. The Tersoff potential was the first to successfully address this problem [23,24]. However, it had the disadvantage that, even after its development, the potential contained only silicon, boron, and nitrogen in addition to carbon, but no hydrogen. It was Brenner who further developed the Tersoff potential for systems containing carbon and hydrogen [25]. Already, the original reactive empirical bond order potential (REBO) successfully treated hydrocarbon molecules alongside various solid carbon structures. The second generation of the reactive empirical bond order potential (REBO-II) contains modified analytical functions for short-range interactions, e.g., dihedral terms, compared to the original REBO [26]. The number of parameters has also been increased to a few dozen and fitted to a larger database. It is important to emphasize that the interaction between two atoms depends not only on the distance between them, but also on the other atoms in their neighborhoods, i.e., it is a many-body interaction. In other words, the bonding of atoms is determined by local bonding neighbors and nonlocal conjugation. The many-body interaction allows the covalent bond to be broken and formed by a corresponding change in atomic hybridization. Some details are given in the Methods section.

In our previous studies, we performed MD simulations of encapsulated free carbon atoms within an (18,0) SWCNT and annealed the system at different temperatures using different interatomic potentials suitable for carbon–carbon interaction [22]. The results showed that the Tersoff potential [23,24], in the absence of hydrogen atoms, is significantly more effective in promoting the formation of GNRs in the tube than the alternative potentials tested. However, while the Tersoff potential is considered an effective choice for simulating graphitization processes within the nanotube, its use in simulations involving hydrocarbon molecules is limited. This limitation is because the Tersoff potential is specifically tailored to interactions between carbon atoms and does not take into account interactions with hydrogen.

Further examination in the preceding study revealed that, during reactions within the confined space of the tube, force fields incorporating long-range interaction terms, such as ReaxFF [27,28] and AIREBO [29,30], were less successful in facilitating ribbon formation inside the tube. Therefore, the REBO-II [26] potential emerged as a promising candidate for simulating the chemical reactions of encapsulated hydrocarbon molecules within the SWCNT. The REBO-II potential, which is a short-range potential derived from the Tersoff potential, supports the interactions of both carbon and hydrogen atoms. In addition, the REBO-II potential was supplemented with the Lennard-Jones (LJ) potential to account for the significant van der Waals forces between the carbon atoms in the tube wall and the carbon atoms inside the tube. The values of the LJ parameters were aligned with those used for carbon atoms in the AIREBO potential.

In this work, we continued our previous MD simulations, but instead of independent carbon atoms, we randomly placed different cyclic hydrocarbon molecules inside an (18,0) carbon nanotube as the initial structure. As mentioned above, the REBO-II potential was used for interactions between all atoms within the nanotube. In our simulations, however, we found that the REBO-II potential with the original parameterization is not suitable for describing the formation of carbon nanoribbons, or any meaningful structure at all, within the carbon nanotube, due to the very slow dynamics of H abstraction. To solve this problem and to speed up the reaction, we used a method that involved increasing the temperature and modifying one of the parameters of REBO-II. As a result, this allowed us to simulate the formation of GNRs inside SWCNTs. The details are discussed in this paper.

This study is structured as follows. First, the methodology used is described. This is followed by a detailed discussion of the results obtained for different cyclic hydrocarbon molecules, including C_5_H_5_ (as a model system for ferrocene), C_6_H_6_ (benzene), C_20_H_12_ (perylene), and C_24_H_12_ (coronene), which are dealt with separately in the results section. The discussion starts with C_5_H_5_, representing a component of ferrocene, to investigate the performance of the REBO-II potential on the C_5_H_5_@(18,0) system. In order to further explore the carbon–carbon (C-C), hydrogen–hydrogen (H-H) and carbon–hydrogen (C-H) interactions, simulations have also been performed for C_5_@(18,0) and (C_5_+2.5H_2_)@(18,0) systems. Subsequently, in Section 3.2, we present the results for C_6_H_6_, which can be considered as the simplest unit of polyaromatic hydrocarbons (PAHs), followed by an investigation of the (C_6_+3H_2_)@(18,0) scenario. The discussion concludes in Section 3.3 with the presentation of results for encapsulated perylene (C_20_H_12_) and coronene (C_24_H_12_) molecules.

## 2. Methods

In this study, all molecular dynamics simulations were performed with the open-source software LAMMPS (MPI v1.0: LAMMPS MPI STUBS for LAMMPS version 2, August 2023) [31]. LAMMPS’s flexibility in handling different types of interatomic potentials such as Tersoff, AIREBO, REBO-II, and ReaxFF, allows it to simulate a broad range of phenomena in hydrocarbon systems, from the phase behavior and mechanical properties of polymers to the dynamics of complex biological membranes and the reactive synthesis of carbon-based materials.

The selection of the REBO-II potential was influenced by our prior research, as previously discussed. Furthermore, Tomas et al. assessed the efficacy of six prevalent carbon potentials in LAMMPS, among them REBO-II, for their capability to characterize amorphous carbons and the graphitization procedure [19,20]. Their findings indicated that the REBO-II potential facilitated a 90% sp^2^ bonding configuration in carbon atoms following the annealing of amorphous carbons at low densities, affirming the potential’s effectiveness in GNR synthesis.

The REBO-II potential is a method for calculating the total chemical binding energy ($E_{b}$) of a system, inspired by the chemical pseudopotential theory originally formulated by Abell [26]. This potential is particularly designed to model the interactions within carbon-based systems and is characterized by its focus on nearest-neighbor interactions. The total binding energy ($E_{b}$) is expressed as a combination of repulsive ($V_{R}\left(r\right)$) and attractive ($V_{A}\left(r\right)$) terms between pairs of nearest neighbor atoms:

(1) $$E_{b}=\sum_{i}\sum_{j>i}\left[V_{R\;}\left(r_{ij}\right)-{\bar{b}}_{ij}V_{A}\left(r_{ij}\right)\right]$$

(2) $$V_{R}\left(r\right)=f^{c}\left(r\right)(1+Q/r)Ae^{-\alpha r}$$

(3) $$V_{A}\left(r\right)=f^{c}\left(r\right)\sum_{n=1,3}B_{n}e^{-\beta_{n}r}$$

Both terms are modified with a cut-off function $f^{c}\left(r\right)$ to limit the range of these interactions to nearest neighbors, reflecting the localized nature of covalent bonding. The repulsive term $V_{R}\left(r\right)$ includes an exponential decay function scaled with a parameter A and modified with a prefactor that depends on the distance *r* and a constant *Q*. The attractive term $V_{A}\left(r\right),$ on the other hand, sums over contributions from three different types of hybridizations (sp, sp^2^, and sp^3^) of the carbon atom, each with its own exponential decay characterized by coefficients $B_{n}$ and decay rates $\beta_{n}.$ The bond order term ${\bar{b}}_{ij}$ is a crucial component of the REBO-II potential, encapsulating the dynamic nature of chemical bonding in response to the local environment, including effects such as bond saturation and the presence of conjugated systems. It is calculated as an average and correction term involving the geometry and electronic structure around the bond between atoms *i* and *j*, including terms for radical, conjugate, nonconjugate, double bond, and torsion effects.

(4) $${\bar{b}}_{ij}=\frac{1}{2}\left(b_{ij}+b_{ji}\right)+\pi_{ij}^{RC}+b_{ij}^{DH}$$

(5) $$b_{ij}=[1+\sum_{k\left(\neq i,j\right)}f_{\mathrm{ik}}^{c}\left(r_{ik}\right)G\left(cos\theta_{kij}\right)e^{\alpha_{kij}\left[\left(r_{ij}-R_{ij}^{eq}\;\right)-\left(\;r_{ik}-R_{ik}^{eq}\;\right)\right]}+P_{ij}\left(N_{i}^{C},N_{i}^{H}\right)]-1/2$$

The bond order $b_{ij\;}$ itself is a complex function accounting for the influence of surrounding atoms on the bond’s character, integrating terms for angular dependencies, the difference in ideal bond lengths, and a spline function dependent on the local chemical environment (number of *C* and *H* neighbors). One can find the details in [26].

The REBO-II potential is designed to handle only short-range atomic interactions, lacking the capability to manage long-range interactions. From the findings in our prior research, the unique, confined space within the tube meant that employing long-range interaction potentials like REAXFF and AIREBO resulted in the formation of chain structures inside the tube, aligned parallel to its walls. Conversely, it is crucial to maintain the van der Waals distance between the atoms inside the tube and the tube walls to prevent the inner atoms from approaching the walls closer than the van der Waals threshold of 3.4 Å. To address this, we utilized the hybrid-style command in LAMMPS to specifically apply the Lennard-Jones (LJ) potential between the tube walls and the carbon atoms inside the tube, effectively managing these interactions.

The initial atomic configuration for the molecular dynamics (MD) simulations was created using the PACKMOL software (Version: v20.14.3). The reactant molecules were randomly arranged inside a 3 nm long segment of an (18,0) SWCNT with a diameter of 1.4 nm. The number of guest molecules was chosen to correspond to around 33 C atoms/nm, based on experimental observations [14]. Just for comparison, this density of C atoms corresponds to an armchair nanoribbon containing 7 carbon atoms along the width of the nanoribbon (7-AGNR). Therefore, a 3 nm long section of the tube was filled with 20 C_5_H_5_, 16 C_6_H_6_, 5 C_20_H_12_, or 4 C_24_H_12_ molecules in a random initial arrangement. The simulation box was configured with periodic boundary conditions to ensure the formation of a continuous, long nanotube along the tube’s axis, specifically in the z-direction. To prevent interactions between unit cells in the x and y directions perpendicular to the tube, the simulation box was significantly enlarged in these directions. This modification guaranteed a separation of 50 Å between the centers of adjacent tubes along the x and y axes. After conducting numerous convergence and stability calculations to determine the optimal numerical parameters, we applied the following procedure in all cases: The time step throughout the modelling was set to 0.1 fs. The heating and annealing processes were carried out by applying the NVT ensemble, which adjusts the temperature using a Nosé–Hoover thermostat with the damp factor of 0.01 ps. Initially, the temperature of the system was raised from 300 K to the annealing temperature of 3000 K in a few picoseconds (at a rate of 100 K/ps), and then the system was maintained at this temperature for 1 ns. In order to have a better view of the final geometry qualitatively, we applied the energy minimization over the final geometry after the simulation was finished at 1 ns. As a result, however, the deformation of the tube is easily noticeable during the simulation at various intermediate steps; at 1 nanosecond, the deformation becomes evident by comparing the cross-section of the tube between the initial and final configurations.

During the simulations, the carbon nanotube (CNT) remained flexible and engaged in interactions with guest molecules, supported by both REBO-II and Lennard-Jones (LJ) interatomic potentials. The geometry of atoms during the simulations were visualized using the VMD (Visual Molecular Dynamics, 1.9.1 version) software. To quantitatively track the changes in the structure, we used a self-written Python code that calculates the number of hexagons and the average distance from the best in-plane fit of the atom positions (nonplanarity) every 100 fs during the simulation.

Choosing the right temperature was important for running the simulations. Without H atoms, the fusion of the rings started at 1100 K. However, for hydrocarbon molecules, the dynamics of H abstraction proved to be too slow. Therefore, the effect of increasing the temperature on the process was investigated. In cases without H atoms, the formation of fused rings was already significantly accelerated at 3000 K, as will be seen in the Results section. Above 3000 K, however, the nanotube itself started to lose its stability, and at T > 4000 K, it started to disintegrate. Raising the temperature to speed up the simulation is not unprecedented in the literature. In two recent papers, the authors investigated the graphitization of amorphous carbon using and comparing several different interatomic potentials [19,20]. The temperature was raised high enough to do this, but not so high as to melt the structure. For example, for REBO-II the annealing temperature was 4000 K or 4500 K, depending on the density of carbon atoms.

The big problem arises when we turn to hydrocarbon molecules: the increase in temperature alone was not sufficient for H-containing molecules because of the very slow rate of H abstraction reactions. A second aid was needed here. One possibility would have been to randomly remove some H atoms during the simulation. There are examples of this technique in the literature, which has been successfully applied to the study of benzene combustion [16,17] and acetylene-based growth mechanisms in CVD synthesis of carbon nanotubes [18]. We have chosen a different procedure. Instead of randomly removing H atoms, we weakened the strength of the C-H bond. To do this, it was sufficient to modify one of the many parameters of the many-body potential, the βC-H exponent. There is one important difference between the two methods. In the random removal of H atoms, the H atoms removed are permanently removed from the system. In our method, on the other hand, all H atoms remain in the system; it is a self-regulating process.

## 3. Results and Discussion

One of the most important experimental results on the formation of GNRs inside SWCNTs is the study in which ferrocene complex molecules (Fe(C_5_H_5_)_2_) were introduced into the nanotubes [14]. After annealing at high temperature, combined atomic resolution electron microscopy and resonance Raman spectroscopy measurements were used to detect the presence of GNRs inside the SWCNT (mostly 6-AGNR and 7-AGNR). However, realistic simulations with real ferrocene complex molecules are beyond the scope of this work. Instead, the initial phase of the reaction, including the catalytic role of Fe, was omitted, and for simplicity, the simulation was started with single C_5_H_5_ molecules, which is a fragment of ferrocene.

Another important family of precursors are the polyaromatic hydrocarbon (PAH) molecules such as perylene (C_20_H_12_) or coronene (C_24_H_12_). We continued our studies with these molecules. Before that, however, we investigated the C_6_H_6_ scenario, which is the smallest constituent of PAH molecules. In the following sections, we present our results one by one, in the order given.

### 3.1. C_5_H_5_@SWCNT

**Figure 1:**
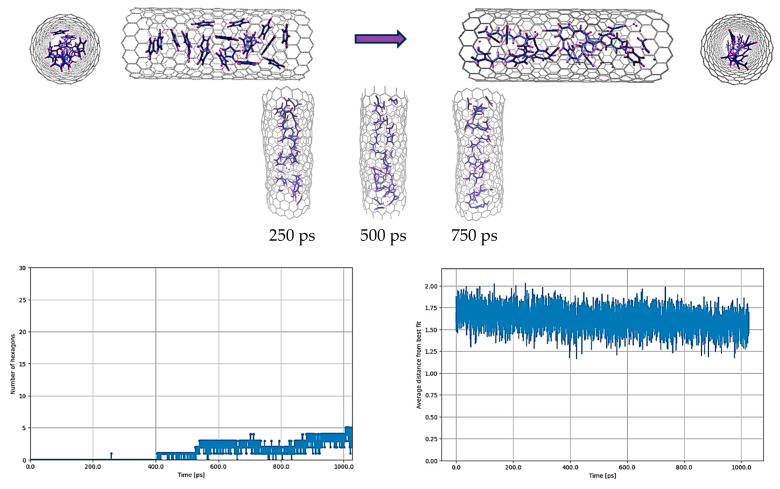
C_5_H_5_@(18,0) with the original parameterization of REBO-II. Here, 20 C_5_H_5_ molecules are encapsulated inside a (18,0) SWCNT with lenght of 3 nm. The top left picture shows the initial random configuration of molecules and the top right one shows the result of simulation at 3000 K after 1 ns. In addition the configurations of atoms at the intermediate steps of 250 ps, 500 ps and 750 ps are shown too. The deformation of the tube is easily noticeable during the simulation at various steps. Due to applying energy minimization to the final geometry of atoms at 1 nanosecond, the deformation becomes evident when comparing the cross-section of the tube between the initial and final configurations. The bottom left diagram shows the number of hexagons and the bottom right one shows the nonplanarity of the structure by determining the average distance (in Å) from the best-fitting plane over the run time in ps.

The MD simulations were started with the original parameterization of the REBO-II potential. Figure 1 shows the result of a simulation in which individual C_5_H_5_ molecules were encapsulated in a (18,0) SWCNT at 3000 K for 1 nanosecond. The number of C_5_H_5_ molecules was 20 per 3 nm. At this elevated temperature, it was observed that many C-C bonds were broken, leading to some fusion of carbon atoms. The resistance of the C-H bonds to breakage, however, prevented the complete fusion of carbon atoms, thus preventing the graphitization process. Very few hexagons were formed, and no planar structure was created as can be seen in Figure 1. The average distance of the atoms from the plane fitted via the least squares method during the simulation practically preserved the nonplanarity value obtained for the initial random structure.

It should be noted that at lower temperatures a less graphene-like structure was formed, due to the C-H bonds being too strong. In fact, we started with lower annealing temperatures, gradually increasing them from 1000 K onwards, and in the end the C-H bonds started to break at around 3000 K. However, further investigations, even at temperatures exceeding 4000 K, revealed that such high temperatures were not only ineffective in significantly breaking the C-H bonds, but also resulted in the disruption of the C-C bonds within the SWCNT. Consequently, this led to the destabilization of the entire system, which is an undesirable outcome. This is why the simulations were all performed at 3000 K.

In an effort to elucidate the behavior of C_5_H_5_, the simulation was repeated under two additional scenarios. In one case, there are no hydrogen atoms, but only C_5_ units are placed in the nanotube. This approach enabled an examination of whether the REBO-II potential can facilitate appropriate C-C interactions during the simulation. In the other case, a mixture of unbonded C_5_ and H_2_ molecules was placed inside the SWCNT so that the number of C atoms and the number of H atoms were exactly the same. We can think of this as artificially breaking the C-H bonds of C_5_H_5_ to focus on whether or not the system will be able to form a ribbon by skipping the initial C-H bond breakage. All simulations were conducted under identical thermodynamic conditions, with the variation lying solely in the different atomic structures filled within the tube.

**Figure 2:**
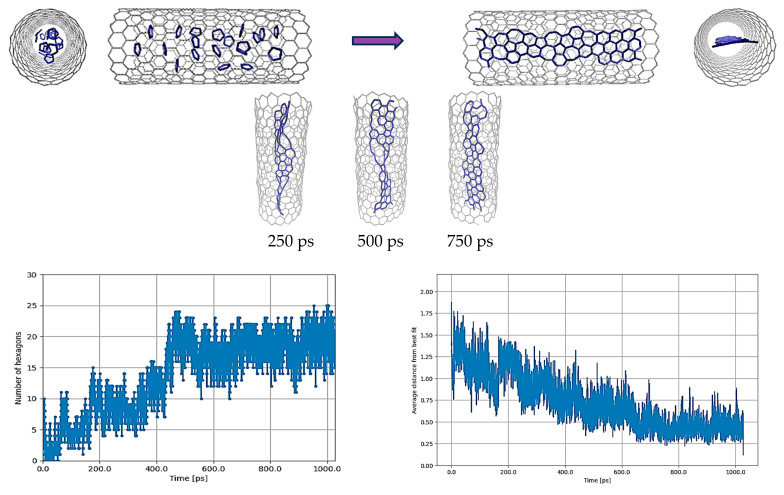
C_5_@(18,0) with the original parameterization of REBO-II. Here, 20 C_5_ structures are encapsulated inside a (18,0) SWCNT with lenght of 3 nm. The top left picture shows the initial random configuration of molecules and the top right one shows the result of simulation at 3000 K after 1 ns. In addition, the configurations of atoms at the intermediate steps of 250 ps, 500 ps, and 750 ps are also shown. The deformation of the tube is easily noticeable during the simulation at various steps. Due to applying energy minimization to the final geometry of atoms at 1 nanosecond, the deformation becomes evident when comparing the cross-section of the tube between the initial and final configurations. The bottom left diagram shows the number of hexagons and the bottom right one shows the nonplanarity of the structure by determining the average distance (in Å) from the best-fitting plane over the run time in ps.

In the scenario devoid of hydrogen atoms, specifically the C_5_@CNT case, the process of heating the molecules and maintaining them at 3000 K for 1 ns resulted in the formation of a ribbon structure, albeit with some defects, as depicted in Figure 2. The initial arrangement of the C_5_ molecules was obtained by simply deleting the hydrogen atoms from the same initial geometry as shown in Figure 1. Figure 2 reveals an increase in the number of hexagons relative to pentagons, alongside the planarity of the final geometry. Although the hexagonal structure exhibited defects, its quality, when compared to the results obtained with C_5_H_5_ under similar conditions, was markedly superior and more closely aligned with experimental observations.

**Figure 3:**
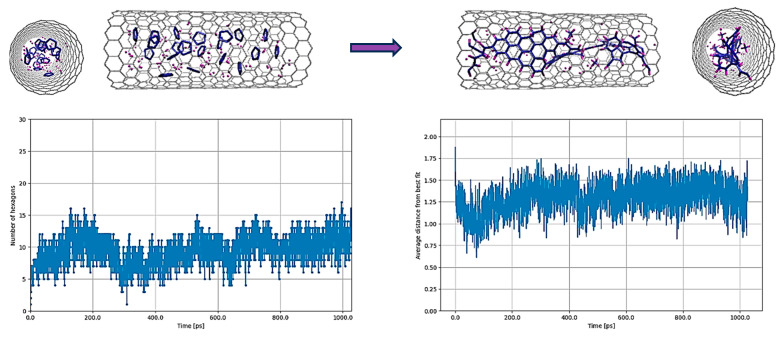
(C_5_+H_2_)@(18,0) with the original parameterization of REBO-II. Here, 50 H_2_ molecules plus 20 C_5_ structures are encapsulated inside a (18,0) SWCNT with lenght of 3 nm. The top left picture shows the initial random configuration of molecules and the top right one shows the result of simulation at 3000 K after 1 ns. The bottom left diagram shows the number of hexagons and the bottom right one shows the nonplanarity of the structure by determining the average distance (in Å) from the best-fitting plane over the run time in ps.

These results suggest that the REBO-II potential can simulate C-C interactions well. However, it appears that the robust C-H interaction within this potential hinders the fusion of C-C bonds (see Figure 1) and, consequently, the formation of GNR structures within the SWCNT, in contrast to the case shown in Figure 2. To investigate the C-H interactions more precisely, the simulation was repeated with the presence of H atoms, albeit employing a different methodology. In this approach, the breaking of C-H bonds from C_5_H_5_ was deliberately omitted. Instead, H_2_ molecules were added randomly to the original starting C_5_ structure of Figure 1, and the resulting new complete starting structure is shown in the upper left of Figure 3.

The bond energies of the C-C and C-H bonds in the hydrocarbon molecules and fragments we studied are close to each other. For example, the REBO-II potential is parameterized so that the C-C bond energy is 3.69 eV for ethane and 5.36 eV for benzene, and the C-H bond energy is typically 4.53 eV [26]. It should be remembered that the bond energy (for both C-C and C-H) depends on the actual structure. The wave function of the electrons in a given bond varies depending on the arrangement of the neighbouring atoms—this is referred to by the expression, empirical bond order, which is also used in the name of REBO. This effect is handled by the REBO potential through the many-body terms.

Due to the close lying C-C and C-H bond energies, there is competition between the formation of C-H bonds and the rearrangement of C-C bonds. We expected that this method would result in a higher degree of C-C fusion compared to the C_5_H_5_ case, which was indeed observed (cf. Figure 1 and Figure 3).

Remarkably, this alternative approach of adding hydrogen molecules led to an increase in the number of hexagonal structures compared to the original C_5_H_5_ situation (cf. Figure 1 and Figure 3). However, this increase is not as large as for molecules containing only carbon (cf. Figure 2 and Figure 3). Additionally, a partially armchair graphene nanoribbon, terminated with hydrogen atoms, was observed in the results, correlating well with experimental findings. While hydrogen passivation appears to be essential and beneficial for achieving more planar and stable graphene nanoribbons, it also implies that once a C-H bond is formed, it tends to remain stable and does not readily break.

Comparing these findings with those observed for C_5_H_5_ highlights the quicker formation of C-H bonds compared to their cleavage, and their resilience against breaking prevents the decomposition of hydrocarbons during reactions within the tube. Similarly, the adverse effect of hydrogen in hindering the formation of carbon ring structures was also observed in DFTB studies conducted by K. Morokuma and coworkers [16,17,18], who managed the average H/C ratio by selectively removing hydrogen atoms throughout their simulation. Essentially, for the breakdown of hydrocarbon molecules and to subsequently enhance carbon graphitization, the potential must be capable of managing the H/C ratio during the reaction, necessitating accurately defined bonds throughout the process.

The simulation results for pure carbon molecules, specifically C_5_ encapsulated in a SWCNT (C_5_@SWCNT), indicate that the parameters governing C-C interactions in the REBO-II potential are adequately defined. However, the presence of hydrogen atoms is a critical factor in the formation of graphene nanoribbons. To facilitate this, it is essential to address the challenge of strong C-H bonds in the reaction of hydrocarbon molecules. However, it appears necessary to modify the parameters related to the C-H bonds. Interestingly, instead of altering the complex many-body terms or parameters in the REBO-II potential (contained in *b_ij_* of Equation (1)) [26], a more efficient approach was found. This approach involves weakening the C-H bond by adjusting a single parameter, β_C-H_, in the $V_{A}\left(r_{ij}\right)$ attractive pair potential term (see Equations (4) and (5)), regardless of the local neighbourhood.

Here, in order to weaken the C-H bond energy, as described at the end of the Methods section, we leave the parameters in the *b_ij_* expressions at their original values and simply decrease the strength of the attractive pair potential by gradually increasing the value of β_C-H_ in $V_{A}\left(r_{ij}\right)$. The original REBO-II version has a β_C-H_ value of 1.4344, corresponding to a bond energy of 4.526 eV for an isolated C-H bond, that is, when *b_ij_* is 1.

**Figure 4:**
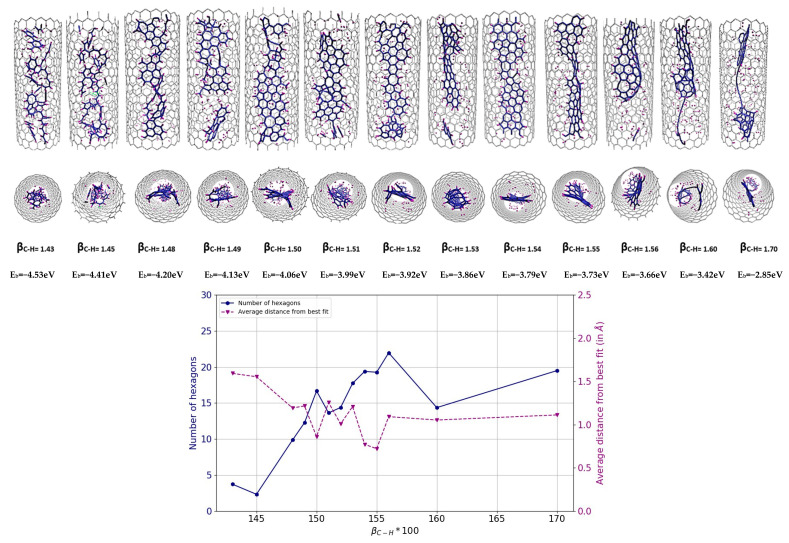
Results of MD simulations of C_5_H_5_@(18,0) using REBO-II potential with different β_C-H_ parameters. A total of 20 C_5_H_5_ molecules are encapsulated inside a (18,0) SWCNT with lenght of 3 nm. The simulations were performed at 3000 K for 1 ns. The top picture shows how the final geometry of the system inside the tube changed with the weakening of the C-H bond energy in the REBO-II potential. The bottom diagram shows the number of hexagons (left axis, blue) and the nonplanarity (right axis, magenta) of the final structure as a function of β_C-H_.

Figure 4 illustrates the variation of the final result as a function of different β_C-H_ values. From the analysis of the diagrams and images presented, it can be seen that the formation of nanoribbons occurs at a β_C-H_s value between 1.50 and 1.55, with the optimum around 1.54–1.55 (corresponding to a bond energy of 3.8–3.7 eV). At these specific values, the number of hexagons reaches a high value, while, simultaneously, the nonplanarity reaches a minimum. In this range, the formation of a ribbon structure within the tube can be successfully achieved. Conversely, a further reduction in the C-H bond energy leads to a decrease in the hydrogen passivation of carbon atoms at the edges, which makes the system more prone to form a more closed system, even an inner tube. This phenomenon is noticeable in the results for β_C-H_ values greater than 1.55, where an increased local density of carbon atoms inside the tube is observed, indicative of the initial stages of inner tube formation. It is important to note again that the density of carbon atoms within a (18,0) CNT corresponds to the formation of a GNR with a width of approximately 7 Å.

**Figure 5:**
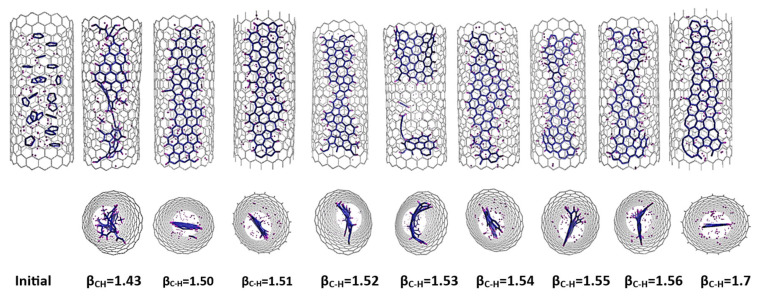

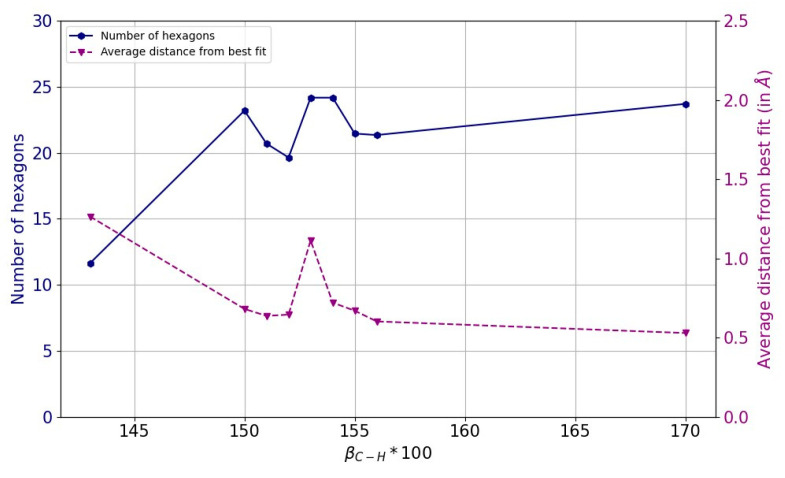
Results of MD simulations of (C_5_+H_2_)@(18,0) using REBO-II potential with different β_C-H_ parameters. A total of 50 H_2_ molecules plus 20 C_5_ structures are encapsulated inside a (18,0) SWCNT with lenght of 3 nm. The simulations were performed at 3000 K for 1 ns. The top picture shows how the final geometry of the system inside the tube changed with the weakening of the C-H bond energy in the REBO-II potential. The bottom diagram shows the number of hexagons (left axis, blue) and the nonplanarity (right axis, magenta) of the final structure as a function of β_C-H_.

We return to the case of C_5_+H_2_ to see whether the results are consistent with those of C_5_H_5_ for the modified parameters of β_C-H_ or not. However, it should be kept in mind that these are different cases, and in the case of C_5_+H_2_, even using REBO-II with the original parameterization, the simulation results in a partially ribbon structure (remember Figure 3). Simulations showed that the REBO-II potential performs well with the modified version when β_C-H_ values are between 1.51 and 1.56; see Figure 5. An optimum is achieved when β_C-H_ is around 1.54. This shows that the results for C_5_H_5_ and C_5_+H_2_ are consistent with each other.

### 3.2. C_6_H_6_@SWCNT

In our study, we have further investigated the activation of C-H bonds within the REBO-II potential, specifically in the context of benzene molecules (C_6_H_6_) encapsulated in a SWCNT. This investigation entailed an analysis of the results obtained from simulations of C_6_H_6_ encapsulated in a SWCNT (C_6_H_6_@SWCNT), and a mixture of C_6_ and H_2_ in a SWCNT ((C_6_ + H_2_)@SWCNT), following a similar approach as before.

**Figure 6:**
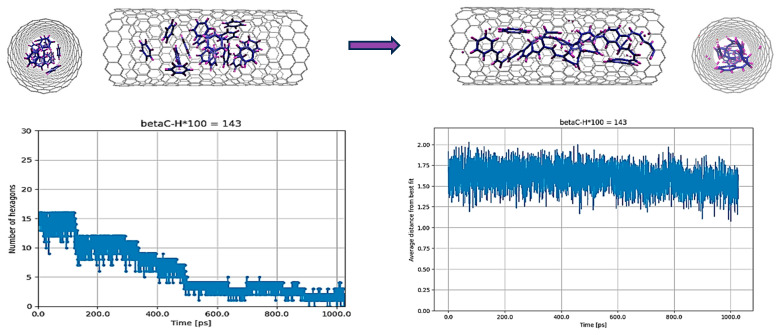
C_6_H_6_@(18,0) with the original parameterization of REBO-II. Here, 16 C_6_H_6_ molecules are encapsulated inside a (18,0) SWCNT with lenght of 3 nm. The top left picture shows the initial random configuration of molecules and the top right one shows the result of simulation at 3000 K after 1 ns. The bottom left diagram shows the number of hexagons and the bottom right one shows the nonplanarity of the structure by determining the average distance (in Å) from the best-fitting plane over the run time in ps.

As illustrated in Figure 6, using the original parameters of REBO-II, at a temperature of 3000 K, the interactions of C_6_H_6_ molecules inside the nanotube mainly affected the C-C bonds, while most of the C-H bonds remained stable. This stability was akin to that observed in the C_5_H_5_@CNT case, which did not yield any notable products.

**Figure 7:**
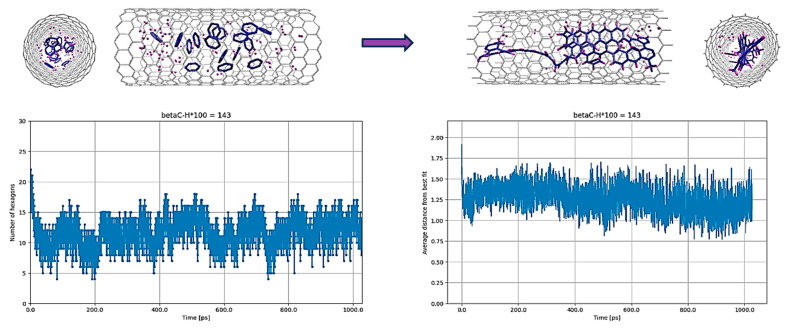
(C_6_+H_2_)@(18,0) with the original parameterization of REBO-II. Here, 48 H_2_ molecules plus 16 C_5_ structures are encapsulated inside a (18,0) SWCNT with lenght of 3 nm. The top left picture shows the initial random configuration of molecules and the top right one shows the result of simulation at 3000 K after 1 ns. The bottom left diagram shows the number of hexagons and the bottom right one shows the nonplanarity of the structure by determining the average distance (in Å) from the best-fitting plane over the run time in ps.

In contrast, the scenario involving a mixture of C_6_ and H_2_ inside the nanotube ((C_6_ + H_2_)@SWCNT) gave a different result. In this case, the hydrogen passivation of carbon atoms at the edges together with the fusion of the C-C bonds at 3000 K led to the formation of a graphene nanoribbon (GNR), albeit not of very good quality, which shows some torsion as shown in Figure 7. This finding highlights the important role of hydrogen in changing the structural dynamics and end products in such simulations.

**Figure 8:**
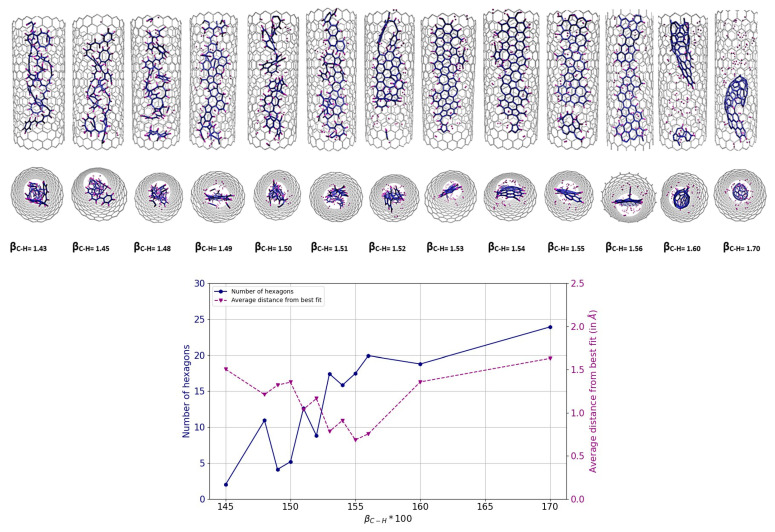
Results of MD simulations of C_6_H_6_@(18,0) using REBO-II potential with different β_C-H_ parameters. A total of 16 C_6_H_6_ molecules are encapsulated inside a (18,0) SWCNT with lenght of 3 nm. The simulations were performed at 3000 K for 1 ns. The top picture shows how the final geometry of the system inside the tube changed with the weakening of the C-H bond energy in the REBO-II potential. The bottom diagram shows the number of hexagons (left axis, blue) and the nonplanarity (right axis, magenta) of the final structure as a function of β_C-H_.

For C_6_H_6_@SWCNT, we performed the same series of simulations with the modified β_C-H_ values as before for C_5_H_5_@SWCNT. Figure 8 illustrates the results, which are similar to those obtained earlier for C_5_H_5_@SWCNT. A ribbon-like structure was formed in the sharp β_C-H_ range between 1.53 and 1.56.

**Figure 9:**
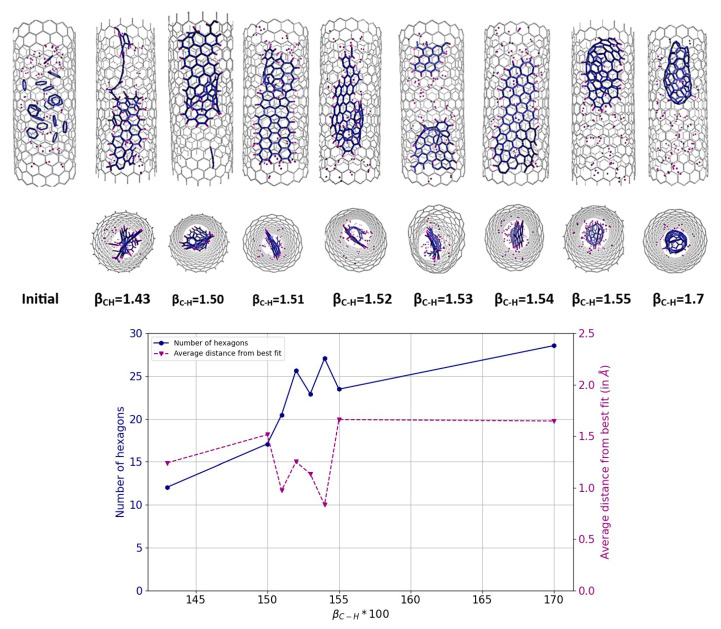
Results of MD simulations of (C_6_+H_2_)@(18,0) using REBO-II potential with different β_C-H_ parameters. A total of 48 H_2_ molecules plus 16 C_6_ molecules are encapsulated inside a (18,0) SWCNT with lenght of 3 nm. The simulations were performed at 3000 K for 1 ns. The top picture shows how the final geometry of the system inside the tube changed with the weakening of the C-H bond energy in the REBO-II potential. The bottom diagram shows the number of hexagons (left axis, blue) and the nonplanarity (right axis, magenta) of the final structure as a function of β_C-H_.

Similar to the C_5_+H_2_ vs C_5_H_5_ case, we also checked whether the C_6_+H_2_ results are consistent with the C_6_H_6_ results for the modified parameters of β_C-H_ or not. Figure 9 illustrates the variation of the final result as a function of different β_C-H_ values. Ribbon formation occurs at β_C-H_s values between 1.51 and 1.54. As can be seen, the formation of a ribbon structure in this β_C-H_ range is similar to the C_5_+H_2_ case. There is one exception that deserves some discussion. For β_C-H_ = 1.55 (and higher values) the difference is striking. For C_6_+H_2_, a fullerene-like structure is formed at β_C-H_ = 1.55, while for C_5_+H_2_, the structure formed is closer to a nanoribbon at the same β_C-H_. The simulation has been examined in detail as a function of time, and it can be seen from the movie that first only the fusion of the C atoms occurs, followed by the passivation of the remaining free carbon atoms via hydrogen atoms.

As we know, the C-C bond in the hexagonal structure is stronger than the C-C bond in a pentagon; so, for the same value of β_C-H_, the difference between the bond energies of C-C and C-H in the different cases of C_5_+H_2_ and C_6_+H_2_ may be slightly different. For example, in the case of (C_6_+H_2_)@SWCNT with β_C-H_ = 1.55, the strong C-C bonds compared to the C-H bonds cause all carbon atoms to fuse together first, and then the H atoms end up bonding to the unsaturated carbon atoms at the edges of the final carbon structure formed in the tube (see Figure 9). Because of the confined space, the carbon structure must also be curved. While in the case of (C_5_+H_2_)@SWCNT, the strength of the C-C and C-H bonds are closer to each other (in fact, this C-C bond is slightly weaker than the C-H bond) and therefore compete with each other. This is the reason why C-H bonds do not allow the formation of a cage-like structure, even at β_C-H_ = 1.70 in the case of (C_5_+H_2_)@SWCNT (see Figure 5). Overall, we can say that the results for C_6_H_6_ and C_6_+H_2_ are consistent with each other.

### 3.3. C_20_H_12_@SWCNT and C_24_H_12_@SWCNT

**Figure 10:**
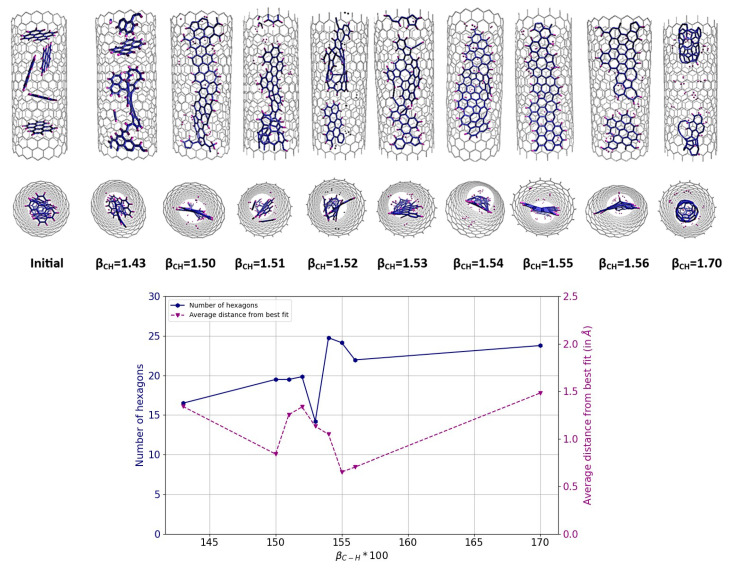
Results of MD simulations of C_20_H_12_@(18,0) using REBO-II potential with different β_C-H_ parameters. A total 5 perylene molecules are encapsulated inside a (18,0) SWCNT with lenght of 3 nm. The simulations were performed at 3000 K for 1 ns. The top picture shows how the final geometry of the system inside the tube changed with the weakening of the C-H bond energy in the REBO-II potential. The bottom diagram shows the number of hexagons (left axis, blue) and the nonplanarity (right axis, magenta) of the final structure as a function of β_C-H_.

**Figure 11:**
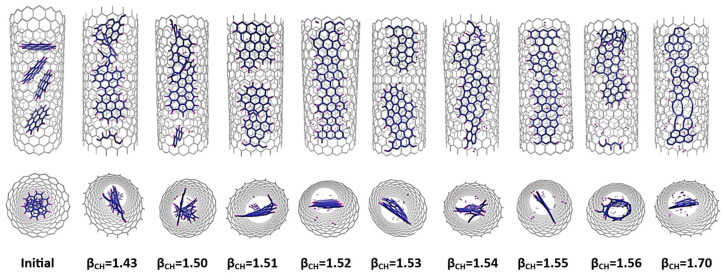

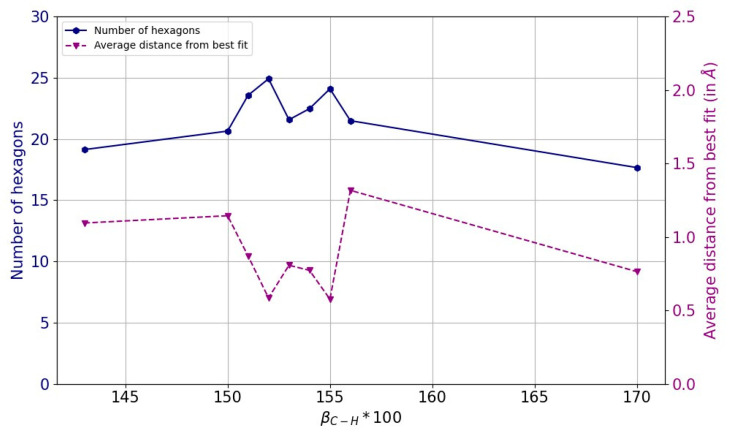
Results of MD simulations of C_24_H_12_@(18,0) using REBO-II potential with different β_C-H_ parameters. A total of 4 of coronene molecules are encapsulated inside a (18,0) SWCNT with lenght of 3 nm. The simulations were performed at 3000 K for 1 ns. The top picture shows how the final geometry of the system inside the tube changed with the weakening of the C-H bond energy in the REBO-II potential. The bottom diagram shows the number of hexagons (left axis, blue) and the nonplanarity (right axis, magenta) of the final structure as a function of β_C-H_.

As mentioned above, polyaromatic hydrocarbon (PAH) molecules, such as perylene (C_20_H_12_) or coronene (C_24_H_12_), are an important family of precursors for the formation of GNRs in SWCNTs. We performed the same series of simulations with the modified β_C-H_ values as before for perylene@SWCNT (see Figure 10) and for coronene@SWCNT (see Figure 11). The results are similar to those obtained earlier for C_5_H_5_@SWCNT and for C_6_H_6_@SWCNT. Both perylene and coronene formed a ribbon-like structure for increased β_C-H_ values similar to those observed for C_5_H_5_ and C_6_H_6_. Specifically, a β_C-H_ range of 1.54 to 1.56 was observed for perylene, with an “optimum” of 1.55, while for coronene the interval and optimum were slightly lower, 1.51–1.55/1.52.

In summary, the following can be said for each of the systems we have studied. The MD simulation with the original parameterization of the REBO-II potential does not lead to GNRs in any case. However, by varying a single parameter, β_C-H_, which determines the C-H bond energy, GNRs are observed in all cases. Importantly, this requires essentially the same β_C-H_ range in all cases. Although the result for a given system may depend somewhat on the initial geometry, this has not been investigated separately, as the fact that the corresponding β_C-H_ for six different systems was obtained in practically the same range seems quite convincing. This is summarized in Table 1. The optimal β_C-H_ that gives the best GNR is almost identical in all cases. In addition, in this optimal range, the final H/C ratio decreased significantly, and, consequently, the final number of C-C bonds increased. Note that this H/C ratio is an “effective” value which is defined as the ratio of the number of H atoms in C-H bonds to the total number of C atoms. Apart from coronene, the final H/C ratio is the same in all cases, with an H/C ratio of 0.30 after 1 ns of MD simulation. For the coronene precursor, the final H/C ratio is lower, 0.26, due to the higher initial number of C-C bonds and the lower initial number of C-H bonds. As we expected, fusing of coronene molecules happened at a lower C-H bond energy of −3.86 eV. Precursor decomposition and hydrogen transfer occur at nearly identical C-H bond energies of −3.76, −3.73, and −3.73 eV for C_5_H_5_, C_6_H_6_, and C_20_H_12_ precursors, respectively, which corresponds to the R-H bond dissociation energy of −3.73 eV found experimentally for R = C_6_H_5_CH_2_—(benzyl radicals) [32].

For the calculation of the bond energy of C-H, only the value of β_C-H_ was changed; for the other parameters, the original REBO-II values were used and the bij = 1 was assumed. The original βC-H = 1.434 gives 4.526 eV for the C-H bond energy.

## 4. Conclusions

Recent experimental results show that by introducing cyclic hydrocarbon molecules inside carbon nanotubes they can be transformed into nanoribbons, depending on the experimental conditions. It is surprising that we could not find any work in the literature in which MD simulations of GNR production, or more generally of any chemical reactions within SWCNTs, have been performed, and we wanted to fill this gap.

We carried out molecular dynamics simulations with several cyclic hydrocarbon molecules encapsulated in a 1.4 nm diameter single-walled carbon nanotube. Various molecules were randomly placed in a (18,0) carbon nanotube as a starting structure. The molecules were as follows: C_5_H_5_ (as a fragment of ferrocene), C_5_, C_5_+H_2_; C_6_H_6_ (benzene), C_6_, C_6_+H_2_; C_20_H_12_ (perylene); and C_24_H_12_ (coronene). The simulations were performed using the REBO-II potential of the LAMMPS software package (MPI v1.0: LAMMPS MPI STUBS for LAMMPS version 2, August 2023), supplemented with a Lennard-Jones potential between the nanotube wall atoms and the inner atoms. We found that in the presence of hydrogen, the REBO-II potential with the original parameterization is not suitable for describing the formation of carbon nanoribbons, or any meaningful structure at all, within the carbon nanotube. The reasons are probably complex. The original parameterization was fitted to the near-equilibrium properties of different hydrocarbon systems (bond length, bond energies, force constants, and bulk elastic properties), while here we investigated dynamic processes and reactions starting from a far-from-equilibrium state, and under unconventional conditions: in the confined space inside a nanotube.

We attempted to reproduce the experimental observations of nanoribbon formation inside SWCNTs. However, the slow dynamics of H abstraction posed a problem. Therefore, we weakened the strength of the C-H bonds to allow the abstraction of H atoms, in a self-regulated manner. Instead of trying to modify the complex many-body expressions of the REBO-II potential, we found a more efficient approach. The strength of the C-H interaction was reduced by modifying only one single parameter, the β_C-H_ in the exponential term of the attractive pair potential, independently of the local neighborhood. This simple process finally allowed us to simulate the formation of GNRs. This success also highlighted the importance of the potential’s ability to handle the H/C ratio during the reaction, in line with the results of previous DFT simulations on carbon nanostructures.

This simple yet elegant method is not limited to describing the generation of GNRs. It can open the way to other similar problems, for example the effect of the diameter of the SWCNT, possibly chirality, on the processes. Such studies are planned or partly ongoing.

## Figures and Tables

**Table 1:** Summary of some data for the systems investigated.

Precursor	Initial Number of C-C Bonds	Initial Number of C-H Bonds	Initial H/C Ratio	β_C-H_ (Å^−1^) Ranges for GNR Formation	Average C-H Bond Energy (eV)	Final Number of C-C Bond	Final Number of C-H Bonds	Final H/C Ratio	Final Value of Nonplanarity (Å)
20 C_5_H_5_	100	100	1	1.54–1.55	3.76	131	31	0.31	0.75
20C_5_ + 50H_2_	100	0	0	1.51–1.56	3.83	126	30	0.30	0.70
16 C_6_H_6_	96	96	1	1.53–1.56	3.73	131	29	0.30	0.70
16C_6_ + 48H_2_	96	0	0	1.51–1.54	3.89	124	29	0.30	0.82
5 C_20_H_12_	120	60	0.60	1.54–1.56	3.73	129	30	0.30	0.66
4 C_24_H_12_	120	48	0.50	1.51–1.55	3.86	124	25	0.26	0.60

## Data Availability

The data that support the findings of this study are available from the corresponding authors upon reasonable request.
